# Contemporaneous International Asthma Guidelines Present Differing Recommendations: An Analysis

**DOI:** 10.1155/2016/3085065

**Published:** 2016-06-23

**Authors:** Samir Gupta, Emily Paolucci, Alan Kaplan, Louis-Philippe Boulet

**Affiliations:** ^1^Department of Medicine, Division of Respirology, University of Toronto, Toronto, ON, Canada M5S 1A8; ^2^The Keenan Research Centre in the Li Ka Shing Knowledge Institute of St. Michael's Hospital, Toronto, ON, Canada M5B 1T8; ^3^Department of Kinesiology, McMaster University, Hamilton, ON, Canada L8S 4L8; ^4^University of Toronto, Toronto, ON, Canada M5S 1A8; ^5^Family Physician Airways Group of Canada, Edmonton, AB, Canada T5X 4P8; ^6^Institut Universitaire de Cardiologie et de Pneumologie de Québec, Université Laval, Québec, QC, Canada G1V 4G5

## Abstract

*Background*. Several international groups develop asthma guidelines. Conflicting recommendations across guidelines have been described in several disease areas and may contribute to practice variability. Accordingly, we compared the latest Canadian Thoracic Society (CTS) asthma guideline with contemporaneous international asthma guidelines to evaluate conflicting recommendations and their causes.* Methods*. We identified the latest CTS asthma guideline update (2012) and the following societies which also updated their guidelines in 2012: the British Thoracic Society and Scottish Intercollegiate Guidelines Network and the Global Initiative for Asthma. We compared these three guidelines on (1) key methodological factors and (2) adult pharmacotherapy recommendations.* Results*. Methods used and documentation provided for literature search strategy and dates, evidence synthesis, outcomes considered, evidence appraisal, and recommendation formulation varied between guidelines. Criteria used to define suboptimal asthma control varied widely between guidelines. Inhaled corticosteroid dosing recommendations diverged, as did recommendations surrounding use of budesonide/formoterol as a reliever and controller and recommendations in the subsequent step.* Conclusions*. There are important differences between recommendations provided in contemporaneous asthma guidelines. Causes include differences in methods used for interpreting evidence and formulating recommendations. Adopting a common set of valid and explicit methods across international societies could harmonize recommendations and facilitate guideline implementation.

## 1. Introduction

Asthma is the third most common chronic disease in adults, affecting 8.1% of the population, or 2.4 million Canadians, and increasing in prevalence [1, 2]. Annually, 150 000 emergency room visits and 60 000 hospital admissions in Canada are attributable to asthma [2], with an economic burden of $1.8 billion in 2011 [3] and a disproportionate cost attributable to patients with poorly controlled symptoms [4].

Effective therapies for asthma exist and well-controlled asthma is achievable in most patients. Several international groups have developed and regularly update evidence-based guidelines aimed at improving asthma control and outcomes. The Canadian Thoracic Society (CTS) published the first Canadian Asthma Guideline in 1990, the US National Heart, Lung and Blood Institute (NHLBI) first issued an asthma guideline in 1991, the Global Initiative for Asthma (GINA) has produced guidelines since 1995, and the British Thoracic Society (BTS) and Scottish Intercollegiate Guidelines Network (SIGN) produced their first joint British guideline in 2001.

However, despite these high-quality guidelines, over half of the patients with asthma remain poorly controlled [5–8]. This has been found to be attributable both to poor patient adherence and to gaps between guideline-recommended care and actual care, across jurisdictions [7, 9, 10]. In fact, the existence of multiple guidelines may result in conflicts between guideline recommendations which may contribute to practice variation and possibly even to poor overall guideline uptake [11–13]. Conflicting recommendations across guidelines have been described in areas as diverse as cancer care (e.g., non-small cell lung cancer) [14], cancer screening (e.g., prostate [15] and breast cancer screening [16]), periodic health exam (e.g., adolescent care [17]), and several chronic diseases, including diabetes mellitus [18] and chronic obstructive pulmonary disease (COPD) [19]. These conflicting recommendations have been shown to cause confusion and frustration among users [11, 12, 19] and to decrease clinician and public trust of guidelines [11, 20, 21]. Accordingly, interguideline conflict may be an incremental contributor to poor guideline adherence.

Given the important consequences of conflicting guideline recommendations, we sought to perform a comparative analysis of the latest Canadian Asthma Guideline with contemporaneous asthma guidelines from around the world.

## 2. Methods

We identified the latest CTS asthma guideline, published in April 2012 [22], and the following societies which also published/updated their guidelines in 2012: the BTS/SIGN (January 2012) [23] and GINA (December 2012) [24]. All three are well-respected international guideline groups which provide information on their guideline production methodologies [24–26]. It should be noted that although the GINA group refers to its product as a “document” or “resource,” it is used as a guideline for all intents and purposes.

We compared these guidelines on the following key methodological factors: literature search strategy and dates; evidence synthesis; outcomes considered; evidence appraisal; recommendation formulation; and overlaps in guideline committee memberships.

Next, we analyzed and compared adult pharmacotherapy recommendations provided in each guideline, in the following three areas: (1) criteria for controller initiation and escalation (asthma control criteria); (2) recommendations for therapeutic escalation of baseline medications (for suboptimal control) (“stepping up” therapy); and (3) recommendations for therapeutic deescalation of baseline medications (after achieving good control) (“stepping down” therapy). We did not compare nonpharmacotherapy recommendations, as these were not reviewed in the 2012 CTS guideline. We also believed that the high quality of evidence for pharmacotherapy choices would minimize the chances of expert opinion-related differences between guidelines.

For comparison of controller escalation recommendations, to account for differences in naming conventions for therapeutic “steps” between guidelines, we designated “step 1” as first-line therapy and steps 2–4 as the subsequent sequential therapeutic intensification recommendations. We did not consider steps beyond “step 4” because they applied to only a small percentage of asthma patients with “difficult-to-treat” asthma, and because evidence is less well-established for these steps [27, 28]. Furthermore, where guidelines offered preferred and alternative therapeutic recommendations, we limited our main comparisons to each guideline's preferred treatment recommendations (those explicitly designated as such and/or featured in tables and/or figures). When guidelines referred to a prior guideline version for specific recommendations which were not covered in the current version or for recommendation references, we consulted the relevant prior guideline version.

For each of the above-described content areas, we also documented the level of evidence attached to each recommendation and compared the references used to support each recommendation, between guidelines. When recommendations and supporting references differed between guidelines, we determined whether the observed differences in references could simply be explained by differences in literature search dates between guidelines.

## 3. Results

### 3.1. Methodological Factors (Table 1)

The 2012 CTS guideline was not presented as a comprehensive, independent guideline statement but rather addressed four very specific PICO questions and reiterated recommendations from the 2010 CTS guideline in other areas. However, this guideline did address therapeutic escalations directly, enabling appropriate comparisons to contemporaneous guidelines. Although it did not revisit asthma control definitions, it reiterated prior definitions, which was the case for the other two guidelines as well.

Literature search strategies varied slightly between guidelines. Search dates were similar for BTS/SIGN and CTS guidelines. The BTS/SIGN guideline had the least current literature review (December 2009), whereas the GINA guideline had the most current (June 2012). Methodological approaches, their documentation, and usage of validated tools varied greatly between guidelines.

With respect to guideline committee membership, there were several common members (including several in leadership roles) between both the GINA and BTS/SIGN guidelines and the GINA and CTS guidelines. The GINA Science Committee included one participant who was cochair of the BTS/SIGN Pharmacological Management Committee and on the BTS/SIGN executive and steering group. The GINA Board of Directors included two participants who were on the 2012 and 2010 CTS Guideline committees (including one who was the Chair of the GINA Board of Directors), and the GINA Science Committee included one participant who was on the 2012 and 2010 CTS Guideline committees and one participant who was on the 2010 CTS Guideline committee.

### 3.2. Adult Pharmacotherapy Recommendations

The highest age category to which recommendations were made applicable was similar between guidelines, as follows: BTS/SIGN > 12 years; GINA “adolescents and adults;” and CTS ≥ 12 years (unless otherwise specified for certain recommendations).

#### 3.2.1. Criteria for Controller Initiation and Escalation (Table 2)

All three guidelines presented criteria for suboptimal control; however, these varied considerably. The GINA guideline recommended using a four-week look-back period to characterize “average” control according to criteria, whereas neither the BTS/SIGN nor the CTS guidelines provided a recommended duration for criterion evaluation. These criteria seemed to apply to both initiation and escalation of controller therapy, as none of the guidelines explicitly recommended distinct criteria for each of these situations.

#### 3.2.2. Therapeutic Escalation by Step (Figure 1)

The CTS guideline was unique in that it presented an “Asthma Management Continuum” diagram, suggesting that management should be approached as more of a continuum than as a series of distinct steps. However, specific recommendations for stepwise therapeutic intensifications were also clearly presented in the text and formed the basis for this comparative analysis.

All three guidelines recommended that all patients start therapy with a short-acting inhaled beta-2-agonist used as needed in step 1. For comparative purposes, the most recent citation for this recommendation in each guideline was published prior to the least current literature review (BTS/SIGN, December 2009), as follows: CTS, 2009; BTS/SIGN, 1999; and GINA, 1997. Next, all guidelines recommended inhaled corticosteroids (ICSs) in step 2; however, recommended doses were higher in the BTS/SIGN guideline. The CTS and BTS/SIGN guidelines did not provide specific references for their initial ICS dose recommendation, and the most recent citation for this in the GINA guideline was from 2005. In step 3, all three guidelines recommended addition of a LABA; however, the GINA and BTS/SIGN guidelines also allowed for use of a single inhaler of budesonide/formoterol as a reliever and a controller (SMART), whereas the CTS guideline did not, concluding that there was insufficient evidence to make this recommendation. Most recent citations for recommendations at this step were as follows: CTS, 2011; BTS/SIGN, 2009; and GINA, 2006. Seven of the references used in the CTS guideline for this step were published after the end-date of the BTS/SIGN literature search. Finally, in step 4, the GINA and BTS/SIGN guidelines suggested increasing the ICS dose (with the BTS/SIGN uniquely suggesting to discontinue the LABA if ineffective), whereas the CTS guideline recommended adding a leukotriene receptor antagonist or switching to the SMART approach. Most recent citations for recommendations at this step were as follows: CTS, 2011; BTS/SIGN, 2007; GINA, 2004. Three of the references used in the CTS guideline for this step were published after the end-date of the BTS/SIGN literature search.

#### 3.2.3. Therapeutic Deescalation by Step (Table 3)

Although all three guidelines recommended reducing to the lowest possible therapeutic dose and regimen after achieving good control, only the GINA guideline provided guidance on each sequential deescalation. However, two of these five recommendations provided in the GINA guideline were derived through panel consensus judgment.

## 4. Discussion

We compared the latest Canadian Asthma Guidelines with two other contemporaneous international guidelines and found major differences between guideline methodologies, asthma control criteria, and recommendations for deescalation of therapy and more minor differences (along with several similarities) between recommendations for escalation of therapy. To our knowledge, this is the first detailed comparative analysis of contemporaneous asthma guidelines presented in the literature.

Previous authors have described varying degrees of interguideline variability in COPD and asthma. Iqbal and colleagues [19] described important differences in recommendations for diagnosis, staging, and therapy of COPD between international guidelines. In 2008, a report by Myers [29] described structure and process differences between non-contemporaneous asthma guidelines, including which key areas were addressed, which evidence grading systems were used, and intended target audiences. It also noted disparities in basic content elements such as whether asthma control was defined, the number of levels of asthma severity, and the number of treatment steps in each guideline.However, this study did not assess methods in detail nor compare control criteria and therapeutic recommendations directly. Furthermore, objective guideline development tools and techniques to improve quality and to harmonize guideline production methodologies have been adopted since 2008. For example, both the CTS [25] and BTS/SIGN groups [26] used the AGREE instrument [30] in 2012 guideline production. Such tools would be expected to mitigate obvious causes of guideline divergence such as variability in literature search, evidence synthesis, and evidence appraisal methods.

### 4.1. Reasons for Differences between Asthma Guidelines (Figure 2)

**Figure 1:**
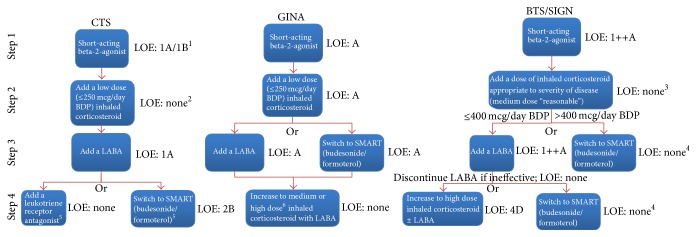
Recommended therapeutic escalation steps and levels of evidence, by guideline. (1) 1A when compared to a LABA; 1B when compared to a LABA/ICS combination. (2) Although it reports an extensive literature review for the recommendation to escalate from a low dose ICS by adding a LABA in step 3 (as opposed to increasing to a medium ICS dose or adding an LTRA), the CTS guideline does not provide a level of evidence nor any reference for initiation at a low ICS dose per se. (3) The BTS/SIGN guideline did not provide a level of evidence nor references for the higher dose approach. In a separate section, authors acknowledged that many patients will benefit more from add-on therapy than from increasing doses above 200 mcg/d BDP. (4) This was an alternative treatment option described in the text only, with no specific level of evidence. (5) The CTS guideline stated that patients uncontrolled on a fixed-dose ICS/LABA should be switched to the SMART approach* in lieu* of increasing the ICS dose of the combination. However, in a separate section, the guideline also noted that “if asthma remains uncontrolled on the combination of an ICS and LABA…consider the addition of an LTRA,” suggesting that either switching to the SMART approach or adding an LTRA would be acceptable. (6) The GINA guideline stated that an increase from medium to high dose provides “relatively little additional benefit,” which seems to contradict the BTS/SIGN approach in both this step and in step 2. See Appendix for a description of the evidence rating system used by each guideline. BDP denotes beclomethasone dipropionate; BTS/SIGN denotes British Thoracic Society/Scottish Intercollegiate Guidelines Network; CTS denotes Canadian Thoracic Society; GINA denotes Global Initiative on Asthma; LABA denotes long-acting beta agonist; LOE denotes level of evidence; SMART denotes Symbicort maintenance and reliever therapy.

Reasons for the differences that we have observed between asthma guidelines are likely multifactorial. One of the most important causes is likely the observed difference in the evidence used (cited) by each guideline group for each recommendation. In turn, there are several possible explanations for this. Search strategies did differ slightly between guidelines; howeve, each was reasonably comprehensive and would not be expected to miss any major studies (Table 1). Search dates differed significantly between guidelines, but as demonstrated in Tables 2 and 3, and in the description of most recent cited references for each therapeutic escalation step (in “Results”), the vast majority of cited studies were published prior to the least current literature review (BTS/SIGN, December 2009), whereby all studies should have been identified by each guideline group. The only exception to this is the handful of studies cited in escalation steps 3 and 4 of the CTS guideline which were published subsequent to the BTS/SIGN literature review date and thus would not have been available to the BTS/SIGN group. However, these would have been available to the GINA group. Accordingly, the differences in evidence used are unlikely to be attributable to differences in literature search strategies and/or dates and are more likely due to differences in evidence interpretation (i.e., synthesis, outcomes considered, and appraisal), methods for which did vary widely between guidelines (Table 1). The importance and impact of these differences in evidence interpretation are demonstrated by our finding that several comparable recommendations were ascribed a certain level of evidence in one guideline and either a different level or no level at all in another guideline (Table 2, Figure 1).

Differences in evidence interpretation might have occurred for both valid and nonvalid reasons. Perfectly valid differences in judgment may have occurred regarding which studies were relevant, the risk of bias in individual studies, and the relative balance of demonstrated benefits and adverse effects. Such differences are an inevitable product from the “human” aspect of evidence interpretation, whereby it may be unrealistic to expect two independently produced guidelines to provide identical recommendations in each area. Interestingly, these guideline committees were not* completely* independent, as there were overlapping guideline committee members and leaders between both GINA and BTS/SIGN and GINA and CTS guidelines. Although one would have expected overlapping committee members to bring common perspectives to the different guideline processes and/or to reconcile major differences in recommendations, this may have been hampered by fundamental differences in process between guidelines. Next, nonvalid factors such as inadequate critical appraisal and/or a failure to consider all relevant outcomes (particularly patient-relevant outcomes) may also have contributed [11]. In all 3 guidelines, evidence selection, synthesis, and appraisal were performed by only a small number (2–4) of committee members, and only the BTS/SIGN group used defined study inclusion and exclusion criteria. Furthermore, although the CTS and BTS/SIGN guidelines used (distinct) validated tools for evidence appraisal, the GINA guideline appears to have employed an informal process (Table 1). Lastly, only the CTS guideline specified which outcomes were considered (including several patient-relevant outcomes). Another concern is the possibility of subjectivity relating to conflicts of interest and the personal values and beliefs of guideline writers [12]. These factors have been shown to contribute to citation selection bias and evidence interpretation bias, even when using objective methods and tools [11, 31]. Financial conflicts of interest aside, “inherent” conflicts such as an author's specialty or publications were recently shown to predict the nature of recommendations surrounding mammography screening, in a study of 12 guidelines and 178 authors [32]. Accordingly, methodological quality does not necessarily correlate with recommendation validity [14].

Differences between methods used for formulating recommendations may also have played a role in the observed variability. The GINA guideline sought full committee consensus, the BTS/SIGN guideline sought unanimity within a small working group, and the CTS guideline sought a majority in a full committee vote (Table 1). In a previous study, Shekelle et al. [33] noted that even with identical committee membership and literature search findings, differences in methods used to combine the literature with expert judgment resulted in important differences in recommendations. For example, in areas of controversy, the informal consensus method produced “lowest common denominator” statements that all panelists agreed upon, but were much less specific than recommendations formulated through a method in which consensus was not mandated.

A myriad of other factors may also have contributed to the variability in recommendations. The relative paucity of available evidence in certain areas, such as the definition of asthma control, required increased use of subjective expert judgments, which are inherently variable [13, 19]. Heterogeneous asthma disease definitions across guidelines and heterogeneous asthma phenotypes across research studies [34] may also have contributed to differences in evidence selection, prioritization, and interpretation between guidelines. Factors such as the availability of health care services and/or medications, and the costs of an intervention in the targeted jurisdiction can also influence how guideline writers view and apply published literature during guideline development [11, 19]. Indeed, the CTS and BTS/SIGN guidelines target audiences in Canada and the United Kingdom, respectively, whereas the GINA guideline targets a global audience. However, we believe that these were unlikely to have been contributors to the differences that we have noted, as relevant medications are widely available, and none of the guidelines explicitly considered costs in their recommendations.

### 4.2. The Consequences of Differences between Asthma Guidelines

The major consequence of disagreement between guidelines is variability in practitioner behavior [13], which may have important clinical implications for asthma evaluation and management. For example, asthma control criteria varied both in terms of recommended cutoffs for elements that were common across guidelines and in terms of which elements were included in each guideline (Table 2). There were also more subtle differences, such as whether daytime symptoms should be counted each* time* they occurred or on each* day* that they occurred (the latter allowing for a single day with several daytime symptom episodes to count only once), and whether rescue beta agonist use should be counted by number of* doses* or by number of* times* the rescue puffer was required (the latter allowing for several consecutive rescue doses at one time to count only once), and whether use prior to exercise was included. The practical impact of these differences was demonstrated in a review of primary care electronic medical records which compared asthma control according to CTS and GINA guidelines and noted interguideline discordance between asthma control ratings in more than half of the patients deemed “in control” by at least one guideline [35]. The criteria resulting in the highest degree of discordance were lung function, absenteeism from school or work, and the frequency of daytime symptoms [35].

There is also a theoretical concern that the observed guideline variability may contribute to the poor uptake of asthma guidelines, which has been noted particularly in the areas of asthma control determination [7] and escalation of therapy [8] (only 39% of Canadian physicians report basing asthma therapy on guideline recommendations) [7]. Asthma guidelines have been noted to be confusing [36] to begin with, and in other diseases, conflicts between major guidelines have been shown to compound confusion, reduce usability, and frustrate practitioners [11, 12, 19]. Qualitative studies suggest that these types of conflicts undermine clinician trust in guidelines, thereby acting as a potential barrier to guideline uptake [11, 20] and contributing to poor guideline adherence [21]. In one study, a positive attitude towards an asthma guideline was more strongly associated with self-reported adherence to the guideline than knowledge of guideline elements themselves [37]. It should be noted that knowledge of asthma guidelines likely remains the most fundamental barrier to uptake [38], and major guideline implementation activities appropriately focus on increasing practitioner knowledge of recommendations. However, our analysis suggests the possibility that attitudinal barriers to asthma guideline acceptance may also exist, particularly in primary care, and merit consideration as a potentially modifiable contributor to poor guideline adherence. However, given that barriers to asthma guideline uptake are diverse, occurring at the level of the patient, the health delivery system, and/or the practitioner [10], quantifying the relative importance, if any, of the observed guideline variability on asthma guideline uptake will require further research.

Finally, studies have found that conflicting recommendations can lead to more test ordering [13] and resulting patient harm [14]. Furthermore, studies suggest that many patients are also aware that conflicting guideline recommendations exist, and this leads to diminished confidence in the medical system and physician recommendations and reduced overall patient adherence [16]. Similarly, conflicting recommendations have been noted to diminish decision makers' faith in guidelines [39], which may in turn limit guideline implementers' ability to enact system-level changes to enable better care.

## 5. Conclusions

We have demonstrated important differences in the basic recommendations surrounding adult asthma care between contemporaneous international guidelines. This has important effects on the consistency of clinical asthma care and may possibly impact overall guideline uptake. Given that this variability appears to have been driven by methodological differences in areas such as evidence interpretation and recommendation formulation, universal acceptance and application of a single, transparent, and explicit set of standards for these processes would likely reduce the observed differences [14, 49]. A common methodological approach to ensure detailed peer review of asthma guidelines by primary care practitioners might also uncover areas where interguideline conflicts are particularly impactful to end-users. Such methodological changes would require a concerted effort for cooperation between the leaders of various international asthma guideline organizations. Given the shared goals and collegiality between international asthma experts, as demonstrated by the common membership and leadership across these guidelines, this goal is achievable and should be a priority. Furthermore, as respiratory organizations increasingly turn their attention to the difficult task of guideline implementation [50, 51], congruence across major international guidelines could enable harmonization and collaboration in these knowledge translation initiatives across jurisdictions, thereby increasing their chances of success.

## Figures and Tables

**Figure 2:**
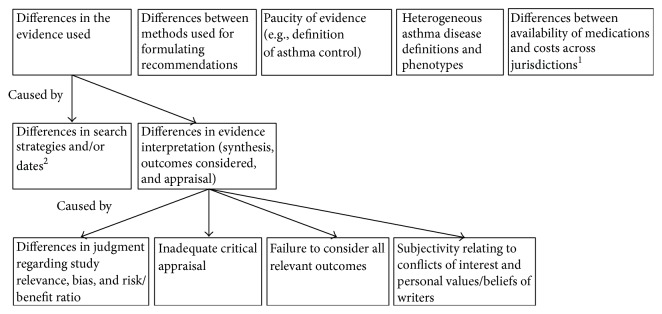
Possible Reasons for observed differences between international asthma guidelines. (1) This is believed to be unlikely to explain the differences between the 3 guidelines in this analysis. (2) This was found to be unlikely to explain the differences between the 3 guidelines found in this analysis.

**Table 1:** Guideline methodologies.

Process	CTS 2012	GINA 2012	BTS 2012
Literature search	Guidelines^1^: MEDLINE, EMBASE, National Guidelines Clearinghouse, CMA Infobase, and GIN (2005 to June 2010) Systematic reviews: MEDLINE, EMBASE, and Cochrane Database of Systematic Reviews (to October 2010) RCTs: MEDLINE, EMBASE, and Cochrane Airways Group Register of Trials (to October 2010)^2^	PubMed (to June 2012) Invitation to “respiratory community” to provide additional abstracts/papers	EMBASE 1980–February 2010 CINAHL 1982–February 2010 MEDLINE 1966–December 2009^3^

Evidence selection	By informal consensus of 3-4-member working groups	By “at least” 2 committee members	By SIGN Executive staff (preliminary), then 1-2 committee members (based on defined inclusion/exclusion criteria)

Evidence synthesis	2 reviewers created data extraction tables	Not provided	SIGN Executive staff created evidence tables after appraisal

Outcomes considered	Need for systemic corticosteroid for exacerbation; ED visits; hospitalizations; time to exacerbation; duration/intensity of exacerbation symptoms; rescue beta2-agonist use; pulmonary function; airway inflammatory markers; quality of life; withdrawals; adverse effects	Not provided	Not provided

Evidence appraisal	AGREE II (for guidelines), AMSTAR (for reviews); Cochrane Risk of Bias instruments (for RCTs)	Each assigned member answered four questions indicating scientific impact^4^	MERGE checklists

Recommendation formulation	Used GRADE scale; anonymous full committee vote rated each recommendation	Used a proprietary evidence scale; full committee consensus or majority (by vote) decided whether/how to change existing recommendations	Used a proprietary evidence scale; the guideline working group unanimously rated each recommendation

^1^The CTS guideline aimed to base recommendations on evidence in existing guidelines, using the ADAPTE process [40] to adapt existing recommendations, where applicable. If no relevant guidelines were found or if guidelines were more than one year old, they searched for systematic reviews; if no relevant reviews were found or if identified reviews' literature searches were more than one year old, they used specific search terms to find randomized controlled trials.

^2^For questions specifically surrounding the efficacy of a single inhaler of budesonide/formoterol as a reliever and a controller (SMART strategy), this search was extended to September 2011.

^3^These were the search criteria used specifically for the pharmacological treatment section.

^4^Questions were not provided in the publication nor on the GINA website.

AMSTAR denotes Assessment of Multiple Systematic Reviews; CMA denotes Canadian Medical Association; ED denotes emergency department; GIN denotes Guidelines International Network; GRADE denotes the Grading of Recommendations Assessment, Development and Evaluation; MERGE denotes Method for Evaluating Research and Guideline Evidence; RCT denotes randomized controlled trial.

**Table 2:** Guideline criteria, levels of evidence, and papers cited for suboptimal asthma control (excluding children).

Criteria	CTS 2012	GINA 2012	BTS 2012
Daytime symptoms	>3 days per week	>2 times per week	>2 times per week

Exacerbations	More than “mild or infrequent exacerbations”^1^	Any exacerbation^2^	Exacerbation requiring oral corticosteroids in the last two years

Reliever requirements	>3 *doses*/week^3^	>2 times/week	>2 times/week

Lung function	FEV1 or PEF <90% personal best	FEV1 or PEF <80% predicted or personal best	No criterion

Diurnal variation	PEF diurnal variation ≥10%^4^	No criterion	No criterion

Sputum eosinophils	Sputum eosinophils ≥2-3%^5^	No criterion	No criterion

Night-time symptoms	≥1 night/week	Any	≥1 night/week

Activity level	Any limitation in physical activity	Any limitation of activities	No criterion

Work/school absence	Any	No criterion	No criterion

Level of evidence^6^	For controller initiation/escalation related to sputum eosinophils: evidence 1B (others not addressed)	For controller initiation related to “symptom frequency” or “periodic worsening”: evidence B (others not addressed)	For controller initiation (all criteria): evidence B
		For controller escalation (any criteria): “This is a working scheme based on current opinion and has not been validated”	For controller escalation (any criteria): none indicated

Papers cited	For controller initiation/escalation: references limited to sputum eosinophil recommendation	For controller initiation related to “symptom frequency” or “periodic worsening”:	For controller initiation (all criteria):
		O'Byrne et al. AJRCCM 2001 [41] Pauwels et al. Lancet 2003 [42] Zeiger et al. Resp Med 2004 [43]	O'Byrne et al. AJRCCM 2001 [41] Pauwels et al. Lancet 2003 [42]
		For controller escalation: none	For controller escalation: none

^1^The CTS guideline also adds: “In…adults presenting with an asthma exacerbation requiring a short course of systemic steroids, daily low- to moderate-dose ICS should be initiated as maintenance long-term therapy” (no level of evidence, no citations provided).

^2^GINA guidelines include a footnote in their “Levels of Asthma Control” table which qualifies uncontrolled asthma with a note that any exacerbation should prompt review of maintenance treatment to ensure that it is adequate.

^3^The CTS guideline includes the use of a reliever to prevent or treat exercise-induced symptoms in its weekly limit.

^4^Listed criterion for good control is PEF diurnal variation <10–15%.

^5^Listed criterion for good control is sputum eosinophils <2-3%; this criterion is only recommended in patients ≥18 years of age with moderate to severe asthma who are assessed in specialist centers.

^6^See Tables 4, 5, and 6 for a description of the evidence rating system used by each guideline.

FEV1 denotes forced expiratory volume in one second; PEF denotes peak expiratory flow.

**Table 3:** Guideline criteria, levels of evidence, and papers cited for controller de-escalation.

Baseline therapy	CTS 2012	GINA 2012	BTS 2012
Medium/high dose ICS plus LABA	No recommendation provided	Reduce the ICS dose by 50% every 3 months and continue the LABA	No recommendation provided
		Level of evidence: B	
		Reference: none provided	

Low dose ICS plus LABA	No recommendation provided	If on a low-dose ICS/LABA combination then discontinue the LABA	No recommendation provided
		Level of evidence: D	
		Reference: none provided	

Medium/high dose ICS alone	No recommendation provided	If the patient is on a medium or high dose ICS alone then reduce the dose of ICS by 50% every three months	Consider decreasing ICS dosing by 25–50% every three months
		Level of evidence: B	Level of evidence: none provided
		References: Hawkins et al. *BMJ* 2003 [44] Powell and Gibson *Thorax *2004 [45] Powell and Gibson *Cochrane Database Syst Rev. *2004 [46]	Reference: Hawkins et al. *BMJ* 2003 [44]

Low-dose ICS alone	If the patient is adequately controlled on low-dose ICS then one should ensure control for 1-2 years prior to stopping ICS therapy altogether Level of evidence: none provided Reference: none provided	If on a low-dose ICS alone and the patient has been controlled for three months then cut down from twice daily to once daily ICS dosing Level of evidence: A References: Boulet et al., *Respir Med. *2006 [47] Masoli et al., and* Respirology *2004 [48] If control is achieved on lowest dose ICS and no recurrence of symptoms occurs for one year then one can discontinue regular ICS therapy Level of evidence: D References: none provided	No recommendation provided

See Appendix for a description of the evidence rating system used by each guideline.

ICS denotes inhaled corticosteroid; LABA denotes long-acting beta agonist.

**Table 4:** CTS guideline.

Grade of recommendation/description	Benefit versus risk and burdens	Methodological quality of supporting evidence	Implications
1A/strong recommendation, high-quality evidence	Benefits clearly outweigh risk and burdens or vice versa	RCTs without important limitations or overwhelming evidence from observational studies	Strong recommendation; it can apply to most patients in most circumstances without reservation

1B/strong recommendation, moderate-quality evidence	Benefits clearly outweigh risk and burdens or vice versa	RCTs with important limitations (inconsistent results, methodological flaws, indirect or imprecise) or exceptionally strong evidence from observational studies	Strong recommendation; it can apply to most patients in most circumstances without reservation

IC/strong recommendation, low-quality, or very low-quality evidence	Benefits clearly outweigh risk and burdens or vice versa	Observational studies or case series	Strong recommendation, but it may change when higher quality evidence becomes available

2A/weak recommendation, high-quality evidence	Benefits closely balanced with risks and burden	RCTs without important limitations or overwhelming evidence from observational studies	Weak recommendation; its best action may differ depending on circumstances and patients' or social values

2B/weak recommendation, moderate-quality evidence	Benefits closely balanced with risks and burden	RCTs with important limitations (inconsistent results, methodological flaws, indirect or imprecise) or exceptionally strong evidence from observational studies	Weak recommendation; its best action may differ depending on circumstances and patients' or social values

2C/weak recommendation, low-quality, or very low-quality evidence	Uncertainty in the estimates of benefits, risks, and burden; benefits, risk, and burden may be closely balanced	Observational studies or case series	Very weak recommendations; other alternatives may be equally reasonable

**Table 5:** BTS/SIGN guideline.

Levels of evidence	
1 ++	High-quality meta-analyses, systematic reviews of RCTs, or RCTs with a very low risk of bias

1 +	Well-conducted meta-analyses, systematic reviews, or RCTs with a low risk of bias

1 −	Meta-analyses, systematic reviews, or RCTs with a high risk of bias

2++	High-quality systematic reviews of case control or cohort studies High-quality case control or cohort studies with a very low risk of confounding or bias and a high probability that the relationship is causal

2+	Well-conducted case control or cohort studies with a low risk of confounding or bias and a moderate probability that the relationship is causal

2−	Case control or cohort studies with a high risk of confounding or bias and a significant risk that the relationship is not causal

3	Nonanalytic studies, for example, case reports and case series

4	Expert opinion

Grades of recommendation	

Note: The grade of recommendation relates to the strength of the evidence on which the recommendation is based. It does not reflect the clinical importance of the recommendation.	

A	At least one meta-analysis, systematic review, or RCT rated as 1++ and directly applicable to the target population; or a body of evidence consisting principally of studies rated as 1+, directly applicable to the target population and demonstrating overall consistency of results

B	A body of evidence including studies rated as 2++, directly applicable to the target population, and demonstrating overall consistency of results; or extrapolated evidence from studies rated as 1++ or 1+

C	A body of evidence including studies rated as 2+, directly applicable to the target population and demonstrating overall consistency of results; or extrapolated evidence from studies rated as 2++

D	Evidence level 3 or 4; or extrapolated evidence from studies rated as 2+

**Table 6:** GINA guideline.

Evidence category	Sources of evidence	Definition
A	Randomized controlled trials (RCTs). Rich body of data.	Evidence is from endpoints of well-designed RCTs that include pattern of findings in the population for which the recommendation is made. Category A requires substantial numbers of studies involving substantial numbers of participants.

B	Randomized controlled trials (RCTs). Limited body of data.	Evidence is from endpoints of intervention studies that include only a limited number of patients, post hoc or subgroup analysis RCTs, or meta-analysis of RCTs. In general, Category B pertains when few randomized trials exist; they are small in size, they were undertaken in a population that differs from the target population of the recommendation, or the results are somewhat inconsistent.

C	Nonrandomized trials. Observational studies.	Evidence is from outcomes of uncontrolled or nonrandomized trials or from observational studies.

D	Panel consensus judgment.	This category is used only in cases where the provision of some guidance was deemed valuable but the clinical literature addressing the subject was insufficient to justify placement in one of the other categories. The panel consensus is based on clinical experience or knowledge that does not meet the above-listed criteria.

## Appendix

### Recommendation Rating Systems

See Tables 4, 5, and 6.

## Abbreviation List

CTS: Canadian Thoracic Society
NHLBI: National Heart, Lung and Blood Institute
GINA: Global Initiative on Asthma
BTS: British Thoracic Society
SIGN: Scottish Intercollegiate Guidelines Network
COPD: Chronic obstructive pulmonary disease
ICS: Inhaled corticosteroid
SMART: Budesonide/formoterol as a reliever and a controller
LABA: Long-acting beta agonist.

## References

[1] Statistics Canada (2012). *CANSIM Table 105-0501 and Catalogue no. 82-221-X*.

[2] (2007). *Life and Breath: Respiratory Disease in Canada, 2007*.

[3] Ontario Lung Association (2011). *Your Lungs, Your Life: Insights and Solutions to Lung Health in Ontario*.

[4] Bedouch P., Marra C. A., FitzGerald J. M., Lynd L. D., Sadatsafavi M. (2012). Trends in asthma-related direct medical costs from 2002 to 2007 in British Columbia, Canada: a population based-cohort study. *PLoS ONE*.

[5] McIvor R. A., Boulet L.-P., FitzGerald J. M., Zimmerman S., Chapman K. R. (2007). Asthma control in Canada: no improvement since we last looked in 1999. *Canadian Family Physician*.

[6] Chapman K. R., Boulet L. P., Rea R. M., Franssen E. (2008). Suboptimal asthma control: prevalence, detection and consequences in general practice. *European Respiratory Journal*.

[7] FitzGerald J. M., Boulet L.-P., Mclvor R. A., Zimmerman S., Chapman K. R. (2006). Asthma control in Canada remains suboptimal: The Reality of Asthma Control (TRAC) study. *Canadian Respiratory Journal*.

[8] Klomp H., Lawson J. A., Cockcroft D. W. (2008). Examining asthma quality of care using a population-based approach. *Canadian Medical Association Journal*.

[9] Mularski R. A., Asch S. M., Shrank W. H. (2006). The quality of obstructive lung disease care for adults in the United States as measured by adherence to recommended processes. *Chest*.

[10] Boulet L.-P., Bourbeau J., Skomro R., Gupta S. (2013). Major care gaps in asthma, sleep and chronic obstructive pulmonary disease: a road map for knowledge translation. *Canadian Respiratory Journal*.

[11] Oxman A. D., Glasziou P., Williams J. W. (2008). What should clinicians do when faced with conflicting recommendations?. *The British Medical Journal*.

[12] Woolf S. H., Grol R., Hutchinson A., Eccles M., Grimshaw J. (1999). Clinical guidelines. Potential benefits, limitations, and harms of clinical guidelines. *British Medical Journal*.

[13] Gordon A. J., Macpherson D. S. (2003). Guideline chaos: conflicting recommendations for preoperative cardiac assessment. *The American Journal of Cardiology*.

[14] Watine J., Friedberg B., Nagy E. (2006). Conflict between guideline methodologic quality and recommendation validity: a potential problem for practitioners. *Clinical Chemistry*.

[15] Gignon M., Braillon A., Chaine F.-X., Dubois G. (2007). Screening for prostate cancer: heterogeneities of recommendations. A French exception?. *Canadian Journal of Public Health*.

[16] Taplin S. H., Urban N., Taylor V. M., Savarino J. (1997). Conflicting national recommendations and the use of screening mammography: does the physician's recommendation matter?. *Journal of the American Board of Family Practice*.

[17] Richmond T. K., Freed G. L., Clark S. J., Cabana M. D. (2006). Guidelines for adolescent well care: is there consensus?. *Current Opinion in Pediatrics*.

[18] Bennett W. L., Odelola O. A., Wilson L. M. (2012). Evaluation of guideline recommendations on oral medications for type 2 diabetes mellitus: a systematic review. *Annals of Internal Medicine*.

[19] Iqbal A., Schloss S., George D., Isonaka S. (2002). Worldwide guidelines for chronic obstructive pulmonary disease: a comparison of diagnosis and treatment recommendations. *Respirology*.

[20] Flottorp S. A., Oxman A. D., Krause J. (2013). A checklist for identifying determinants of practice: a systematic review and synthesis of frameworks and taxonomies of factors that prevent or enable improvements in healthcare professional practice. *Implementation Science*.

[21] Grol R., Dalhuijsen J., Thomas S., In 'T Veld C., Rutten G., Mokkink H. (1998). Attributes of clinical guidelines that influence use of guidelines in general practice: observational study. *The British Medical Journal*.

[22] Lougheed M. D., Lemiere C., Ducharme F. M. (2012). Canadian Thoracic Society 2012 guideline update: diagnosis and management of asthma in preschoolers, children and adults. *Canadian Respiratory Journal*.

[23] British Thoracic Society (2012). *British Guideline on the Management of Asthma: A National Clinical Guideline*.

[24] Global Initiative for Asthma (2012). *Global Strategy for Asthma Management and Prevention: 2012 (Update)*.

[25] Gupta S., Bhattacharyya O. K., Brouwers M. C. (2009). Canadian Thoracic Society: presenting a new process for clinical practice guideline production. *Canadian Respiratory Journal*.

[26] Healthcare Improvement Standard Scotland SIGN 50: A Guideline Developer’s Handbook. http://www.sign.ac.uk/pdf/sign50nov2011.pdf.

[27] Bateman E. D., Boushey H. A., Bousquet J. (2004). Can guideline-defined asthma control be achieved? The gaining optimal asthma control study. *American Journal of Respiratory & Critical Care Medicine*.

[28] Wenzel S. (2005). Severe asthma in adults. *American Journal of Respiratory & Critical Care Medicine*.

[29] Myers T. R. (2008). Guidelines for asthma management: a review and comparison of 5 current guidelines. *Respiratory Care*.

[30] The AGREE Collaboration Appraisal of Guidelines for Research & Evaluation (AGREE) Instrument. http://www.agreetrust.org/.

[31] Shen J., Sun M., Zhou B., Yan J. (2014). Nonconformity in the clinical practice guidelines for subclinical Cushing's syndrome: which guidelines are trustworthy?. *European Journal of Endocrinology*.

[32] Norris S. L., Burda B. U., Holmer H. K. (2012). Author's specialty and conflicts of interest contribute to conflicting guidelines for screening mammography. *Journal of Clinical Epidemiology*.

[33] Shekelle P. G., Kravitz R. L., Beart J., Marger M., Wang M., Lee M. (2000). Are nonspecific practice guidelines potentially harmful? A randomized comparison of the effect of nonspecific versus specific guidelines on physician decision making. *Health Services Research*.

[34] Fajt M. L., Wenzel S. E. (2015). Asthma phenotypes and the use of biologic medications in asthma and allergic disease: the next steps toward personalized care. *Journal of Allergy and Clinical Immunology*.

[35] Dostaler S. M., Olajos-Clow J. G., Sands T. W., Licskai C. J., Minard J. P., Lougheed M. D. (2011). Comparison of asthma control criteria: importance of spirometry. *Journal of Asthma*.

[36] Covvey J. R., Johnston B. F., Wood F., Boyter A. C. (2013). Is the BTS/SIGN guideline confusing? A retrospective database analysis of asthma therapy. *Primary Care Respiratory Journal*.

[37] Tumiel-Berhalter L. M., Watkins R. (2006). The impact of provider knowledge and attitudes toward national asthma guidelines on self-reported implementation of guidelines. *Journal of Asthma*.

[38] Doerschug K. C., Peterson M. W., Dayton C. S., Kline J. N. (1999). Asthma guidelines: an assessment of physician understanding and practice. *American Journal of Respiratory and Critical Care Medicine*.

[39] Hitchen L. (2007). Conflicting guidelines on same topics cause doctors confusion, say MPs. *The British Medical Journal*.

[40] Fervers B., Burgers J. S., Voellinger R. (2011). Guideline adaptation: an approach to enhance efficiency in guideline development and improve utilisation. *BMJ Quality and Safety*.

[41] O'Byrne P. M., Barnes P. J., Rodriguez-Roisin R. (2001). Low dose inhaled budesonide and formoterol in mild persistent asthma: the OPTIMA randomized trial. *American Journal of Respiratory and Critical Care Medicine*.

[42] Pauwels R. A., Pedersen S., Busse W. W. (2003). Early intervention with budesonide in mild persistent asthma: a randomised, double-blind trial. *The Lancet*.

[43] Zeiger R. S., Baker J. W., Kaplan M. S. (2004). Variability of symptoms in mild persistent asthma: baseline data from the MIAMI study. *Respiratory Medicine*.

[44] Hawkins G., McMahon A. D., Twaddle S., Wood S. F., Ford I., Thomson N. C. (2003). Stepping down inhaled corticosteroids in asthma: randomised controlled trial. *British Medical Journal*.

[45] Powell H., Gibson P. G. (2004). Initial starting dose of inhaled corticosteroids in adults with asthma: a systematic review. *Thorax*.

[46] Powell H., Gibson P. G. (2004). High dose versus low dose inhaled corticosteroid as initial starting dose for asthma in adults and children. *Cochrane Database of Systematic Reviews*.

[47] Boulet L.-P., Drollmann A., Magyar P. (2006). Comparative efficacy of once-daily ciclesonide and budesonide in the treatment of persistent asthma. *Respiratory Medicine*.

[48] Masoli M., Weatherall M., Holt S., Beasley R. (2004). Budesonide once versus twice-daily administration: meta-analysis. *Respirology*.

[49] Guyatt G., Gutterman D., Baumann M. H. (2006). Grading strength of recommendations and quality of evidence in clinical guidelines: report from an American College of Chest Physicians task force. *Chest*.

[50] Gupta S., Licskai C., Van Dam A., Boulet L.-P. (2013). Introducing the Canadian Thoracic Society framework for guideline dissemination and implementation, with concurrent evaluation. *Canadian Respiratory Journal*.

[51] Boulet L.-P., FitzGerald J. M., Levy M. L. (2012). A guide to the translation of the Global Initiative for Asthma (GINA) strategy into improved care. *European Respiratory Journal*.

